# Microstructural and mechanical insight into atherosclerotic plaques: an ex vivo DTI study to better assess plaque vulnerability

**DOI:** 10.1007/s10237-022-01671-5

**Published:** 2023-01-18

**Authors:** B. Tornifoglio, R. D. Johnston, A. J. Stone, C. Kerskens, C. Lally

**Affiliations:** 1 Trinity Centre for Biomedical Engineering, Trinity College Dublin, Dublin, Ireland; 2 Department of Mechanical, Manufacturing and Biomedical Engineering, School of Engineering, Trinity College Dublin, Dublin, Ireland; 3 Department of Medical Physics and Clinical Engineering, St. Vincent’s University Hospital, Dublin, Ireland; 4 Trinity College Institute of Neuroscience, Trinity College Dublin, Dublin, Ireland; 5 Advanced Materials and Bioengineering Research Centre (AMBER), Royal College of Surgeons in Ireland and Trinity College Dublin, Dublin, Ireland

**Keywords:** Diffusion tensor imaging, Atherosclerosis, Carotid plaque

## Abstract

Non-invasive microstructural characterisation has the potential to determine the stability, or lack thereof, of atherosclerotic plaques and ultimately aid in better assessing plaques’ risk to rupture. If linked with mechanical characterisation using a clinically relevant imaging technique, mechanically sensitive rupture risk indicators could be possible. This study aims to provide this link–between a clinically relevant imaging technique and mechanical characterisation within human atherosclerotic plaques. Ex vivo diffusion tensor imaging, mechanical testing, and histological analysis were carried out on human carotid atherosclerotic plaques. DTI-derived tractography was found to yield significant mechanical insight into the mechanical properties of more stable and more vulnerable microstructures. Coupled with insights from digital image correlation and histology, specific failure characteristics of different microstructural arrangements furthered this finding. More circumferentially uniform microstructures failed at higher stresses and strains when compared to samples which had multiple microstructures, like those seen in a plaque cap. The novel findings in this study motivate diagnostic measures which use non-invasive characterisation of the underlying microstructure of plaques to determine their vulnerability to rupture.

**Graphic abstract:**

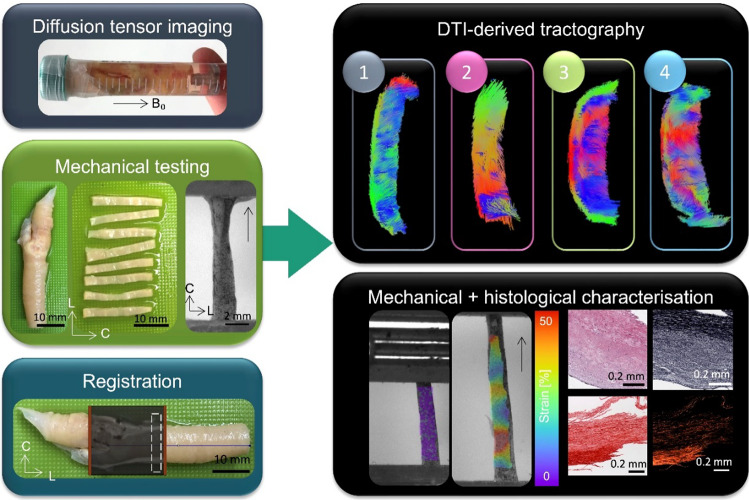

**Supplementary Information:**

The online version contains supplementary material available at 10.1007/s10237-022-01671-5.

## Introduction

There is a growing body of evidence suggesting that the percent stenosis, a diagnostic criterion for atherosclerotic plaque vulnerability established in the 1980s and 1990s (Warlow et al. 1998; Warlow 1991), is not an adequate indicator for measuring the likelihood of a plaque to rupture. While studies have investigated geometric features and remodeling of plaques and their usefulness in determining vulnerability (Ghasemi et al. 2020; Jiang 2020; Wang et al. 2022; Creane et al. 2012), there is a recent acknowledgement that plaque composition and microstructure could better capitulate rupture risk (Naylor et al. 2018; Saba et al. 2019; Kolodgie et al. 2017; Alex et al. 2022). More specifically, the percent stenosis falls short both in low grade stenosed plaques (Wasserman et al. 2005; Holm Nielsen, et al. 2020) and asymptomatic patients (Elhfnawy et al. 2019). A recent study identified that only 62.9% of stroke patients had carotid stenosis, and of that, 54.5% had only mild stenosis (30–49%) (Haque et al. 2022).

Initial studies investigating the composition of atherosclerosis date back to the 1960s (Levene and Poole 1962), while in the last 30 years the mechanical characterisation of plaque tissue has come to the forefront with the aim of better defining rupture risk. A few initial studies of aortic tissue highlighted the compromised mechanical integrity of calcified regions, where they consistently fail at lower strains (Sherebrin et al. 1987; Loree et al. 1994). In carotid and lower limb plaques the stiffness of calcified regions has been linked to micro-computed tomography derived radiographic densities (Cahalane et al. 2018). As increasing studies have been published investigating carotid, coronary, femoral and iliac atherosclerotic plaques – the highly variable nature of these tissues has become more and more apparent (Loree et al. 1994; Born and Richardson 1990; Maher et al. 2009; Lawlor et al. 2011; Holzapfel et al. 2004). Due to the highly complex and dynamic microstructure within these tissues, this variability is to be expected. In the early ‘90s Lendon et al. showed that aortic plaque caps are weaker at locations with high densities of macrophages (Lendon et al. 1993), a conflicting finding to more recent work by Davis et al. in carotid plaques caps (Davis et al. 2016). Specifically, Davis et al. showed that a higher initial stress in the plaque cap is correlated with lower macrophage content and increased collagen content. Johnston et al. showed that collagen orientation, not content, ultimately dictates the mechanical integrity of carotid plaque caps (Johnston et al. 2021). While some studies have used specific microstructural characterisation techniques, such as small angle light scattering (Johnston et al. 2021), Fourier transform infrared and scanning electron microscopy (Mulvihill et al. 2013; Cunnane et al. 2015; Cunnane et al. 2016; Ebenstein et al. 2009), very few have utilised non-invasive, clinically-relevant imaging (Maher et al. 2009; Lawlor et al. 2011; Holzapfel et al. 2004).

Intravascular ultrasound elastography has been used in vivo to estimate the elastic mechanical properties of atherosclerotic plaques in rabbits (Li et al. 2017). Meanwhile, non-invasive carotid elastography on human patients found significantly higher strain rates in vulnerable plaques – identified by magnetic resonance imaging (MRI) (Huang et al. 2016). Additionally, optical coherence tomography has been identified as an invasive imaging modality which can identify plaque features (Yabushita et al. 2002; Subban and Raffel 2020), whereas optical coherence elastography has the potential to identify regions of high strain (Soest et al. 2017). In vivo computed tomography has also been used to classify plaques (Nakao et al. 2021), while ex vivo studies using micro-computed tomography have looked at the mechanical properties around calcified and non-calcified atherosclerotic tissue (Cahalane et al. 2018; Cahalane and Walsh 2021). In comparison to ultrasound, computed tomography and optical coherence tomography, MRI is both non-ionising and offers unparalleled soft tissue contrast. A number of studies have used ex vivo MRI to characterise plaque components (Harteveld et al. 2016; Truong, et al. 2021; Azuma et al. 2020; Meletta et al. 2015; Karmonik et al. 2006; Morrisett et al. 2003; Clarke et al. 2003; Shinnar et al. 1999). These studies utilise different combinations of T1-, T2-, proton-density, and diffusion weighted imaging to gain insight into the plaque composition. The presence of necrotic cores (Meletta et al. 2015; Clarke et al. 2003), lipid cores (Harteveld et al. 2016; Truong, et al. 2021; Meletta et al. 2015; Karmonik et al. 2006; Shinnar et al. 1999; Toussaint et al. 1997), calcifications (Harteveld et al. 2016; Azuma et al. 2020; Meletta et al. 2015; Karmonik et al. 2006; Morrisett et al. 2003; Clarke et al. 2003; Shinnar et al. 1999), fibrous tissue (Harteveld et al. 2016; Truong, et al. 2021; Azuma et al. 2020; Karmonik et al. 2006; Morrisett et al. 2003; Clarke et al. 2003; Shinnar et al. 1999) and fibrous caps (Harteveld et al. 2016; Meletta et al. 2015; Toussaint et al. 1997), inflammation (Truong, et al. 2021; Meletta et al. 2015), hemorrhage (Truong, et al. 2021), red blood cells (Azuma et al. 2020), hemosiderin (Azuma et al. 2020; Meletta et al. 2015), neovascularization (Meletta et al. 2015), thrombus (Karmonik et al. 2006), and solid-state and liquid-lipid (Morrisett et al. 2003) have all been investigated. While knowledge of the composition of atherosclerotic plaques is beneficial in characterising them, there still lacks a direct connection between composition and mechanical integrity. As there is no current clinical imaging technique which correlates composition to mechanics, ex vivo mechanical characterisation of surgically excised specimens must be investigated with an imaging technique which has the potential to be applied clinically.

With previous work pointing to the significance of the alignment of load bearing collagen (Johnston et al. 2021), a non-invasive imaging technique which allows for insight into the overall microstructural alignment of a plaque could aid in characterising its integrity. Diffusion tensor imaging (DTI) is an MRI technique which characterises water diffusion within a tissue and yields insight into the microstructure. While predominantly used clinically in the brain, it has been applied in vivo to myocardial tissue (Stoeck et al. 2021; Mekkaoui et al. 2018) and once in carotid arteries (Opriessnig et al. 2016). To the author’s knowledge, only one study to date by Akyildiz et al. has looked exclusively at unfixed atherosclerotic plaques ex vivo with DTI (Akyildiz et al. 2017). That study showed, for the first time, that a non-invasive imaging technique could characterise the overall microstructural alignment within carotid atherosclerotic plaques. However, these findings were not linked back to the mechanics of the tissue or investigated with respect to composition or specific microstructural components.

The aim of this study is to try to bridge the gap between a clinically relevant imaging technique and mechanical integrity in carotid atherosclerotic plaques. To achieve this, fresh carotid plaques from endarterectomy surgeries were imaged ex vivo with DTI to characterise the microstructure, then subsequently mechanically tested to failure and histologically processed. Altogether, the work presented here seeks to investigate and establish if non-invasive imaging metrics can inform the vulnerability of a plaque and ultimately surpass the percent stenosis as a clinical indicator of plaque rupture risk.

## Methods

### Sample acquisition

Carotid atherosclerotic plaques (n = 7) were obtained from symptomatic carotid endarterectomy patients at St. James’s Hospital Dublin. All patients had a percent stenosis greater than 50%. Ethical approval was obtained from St. James Hospital ethical committee in compliance with the declaration of Helsinki (2016–12 List 47 (4)). Carotid plaques were rinsed in phosphate buffered saline (PBS) to remove residual blood and cryopreserved as previously reported (Johnston et al. 2021). Specifically, a tissue freezing medium with RPMI as the vehicle solution, 1.8 M dimethyl sulfoxide as the permeating cryoprotecting agent, and 0.1 M sucrose as the non-permeating cryoprotecting agent was used (Müller-Schweinitzer 2009). After the PBS wash, samples were placed into room temperature tissue freezing medium and immediately frozen down at a controlled rate of 1 °C/min to -80 °C using a Mr. Frosty (Merck). Samples remained at -80 °C until ex vivo imaging, after which the samples were cryopreserved again using the same tissue freezing medium and controlled freezing rate. Cryopreservation was used to maintain microstructural integrity of the samples between collection, imaging, and mechanical testing. Each sample was cryopreserved twice. 

### Ex vivo imaging

**Fig. 1:**
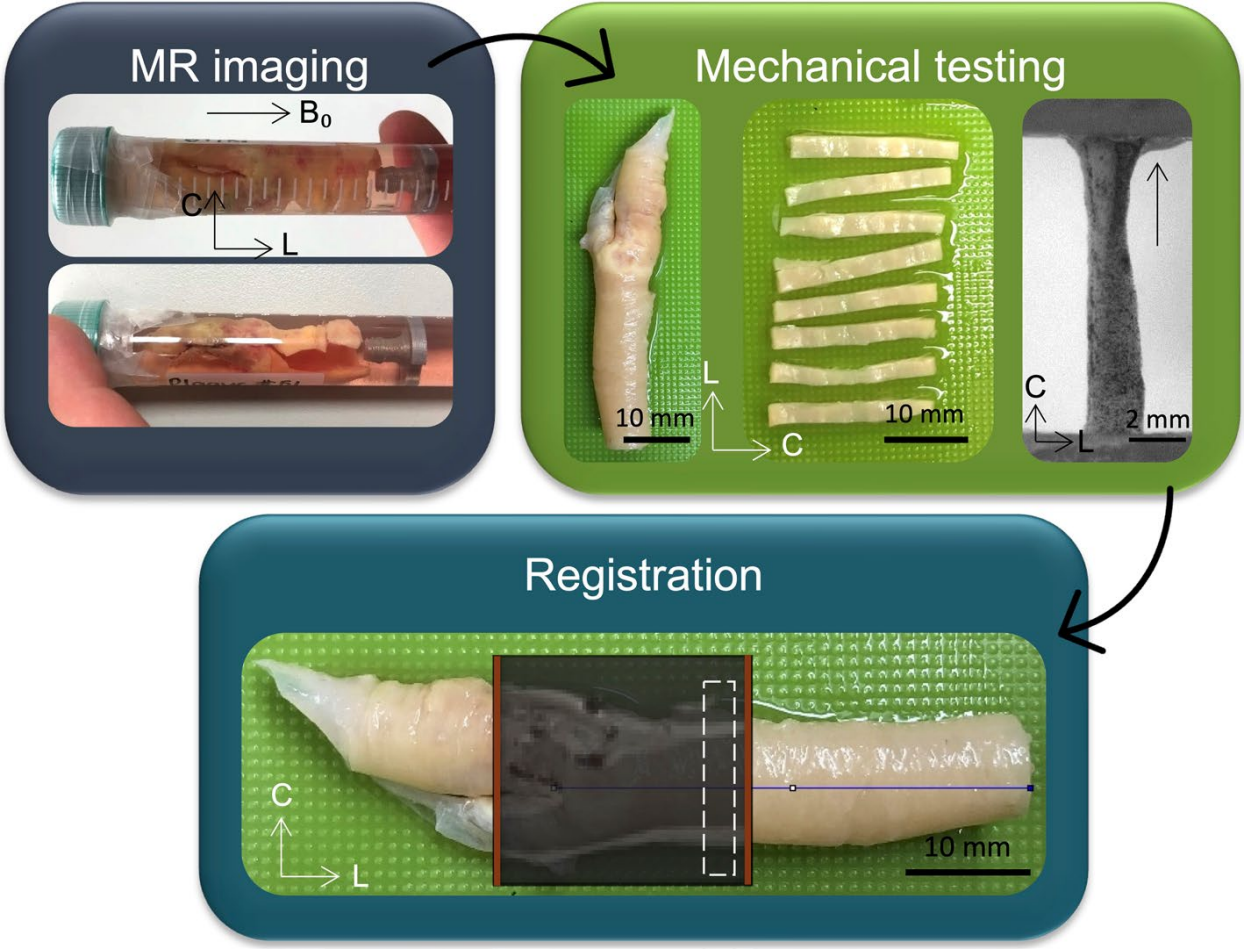
General overview of main methods in this study. Fresh carotid plaques were imaged ex vivo and then circumferential strips were sectioned and uniaxially extended to failure. MR data was then registered to specific mechanically tested strips to facilitate the investigation of DTI-metrics and mechanical properties.

On the day of ex vivo imaging specimens were rapidly thawed in a pre-warmed water bath at 37 °C and rinsed in PBS to remove residual tissue freezing medium. Fresh plaques were secured to a custom-made 3D-printed holder in a 15 ml falcon tube with fresh PBS for imaging, see Fig. 1. All plaques were imaged individually and at ambient room temperature (approximately 25 °C). The longitudinal axis of the plaque was positioned parallel to B_0_ (the main magnetic field) in the scanner (z-axis), with the plane normal to B_0_ the transverse plane (circumferential-radial plane). All imaging was performed in a small-bore horizontal 7 Tesla Bruker BioSpec 70/30 USR system (Bruker Ettlinger, Germany) with a receive-only 8 channel surface coil, birdcage design transmit coil, shielded gradients (maximum strength 770 mT/m) and Paravision 6 software (Bruker, Ettlinger Germany). A conventional 3D-spin echo DTI sequence, previously used on porcine carotid arteries (Tornifoglio et al. 2020) was used. The parameters were as follows: TE/TR: 17.682/1000 ms, image size: 64 × 64 × 64, field of view: 16 × 16 × 16 mm, isotropic resolution: 250 × 250 × 250 μm, b-values: 0, 800 s/mm^2^, 10 b-directions, with fat suppression on and acquisition time: 12 h and 30 min. After imaging, plaques were cryopreserved at -80 °C until mechanical testing.

### Mechanical testing

#### Circumferential strips

Samples were thawed at 37 °C as described in Sect. 2.2 and rinsed in PBS. Circumferential strips (n = 32) were sectioned from the plaques, each with a width of 2 mm, as seen in Fig. 1. A 3D-printed blade holder was used with blades 2 mm apart to ensure all strips were accurately sectioned (Supplementary Fig. 1 (A)). Images of each strip were taken to measure dimensions in ImageJ (Rueden et al. 2017). Three measurements were taken, and mean width and thickness were used for the calculation of the cross-sectional area. Of 32 tested strips, 15 strips were excluded from analysis due to either failure near the grips or inability to be referenced back to MR data. The number of strips from individual plaques are as follows: 5, 3, 1, 4, 1, 2, and 1. Of the 17 strips presented in this study, 14 strips were taken from the plaque within the common and three from plaque in the internal carotid branch. In order to use digital image correlation (DIC), strips were sprayed with a tissue marking dye (Epredia™, Fisher Scientific) using an airbrush (Kkmoon airbrush) compressor (ABEST Single Cylinder Piston Compressor).

#### Uniaxial tensile tests and digital image correlation

Strips were uniaxially extended to failure using a uniaxial test machine (Zwick Z005, Zwick GmbH & Co. Ulm, Germany) with a 20 N load cell (KAP-TC, AST). All tests were performed in a PBS bath at 37 °C and the initial gauge length approximately 8 mm to ensure a 4:1 length to width ratio during testing (Mulvihill and Walsh 2013; Walsh et al. 2014). Strips were gripped between metal grips with velcro and super glue (not placed near the gauge length) and manually tightened without a torque wrench (Supplementary Fig. 1 (B)). The testing protocol included a preload to 0.01 N, after which the force was zeroed and followed by five preconditioning cycles to 5% strain, then extension to failure. All steps were done at a deformation rate of 20 mm/min, equating to approximately 4% the gauge length per second. A DIC system with a two-camera set-up was used (Dantec Dynamics GmbH, Denmark) to track local strain deformations throughout the test, with camera calibration performed prior to testing. Images were acquired from both cameras at a frame rate of 5 Hz. After failure all samples were fixed in 10% formalin for histological processing.

### Histological analysis

After fixation, strips were stepwise dehydrated (Leica TP1020, Semi-enclosed benchtop tissue processor, Germany) and embedded in paraffin wax blocks. Strips were either embedded to get 1) axial cross-sections – such that the lumen was oriented perpendicular to the face of the wax block or 2) with the luminal side of the strip flush with the face of the wax block to achieve cross-sections radially through the wall thickness. The integrity of the strip after mechanical testing dictated which of these options was most feasible; specifically, if it was not possible to orient the samples such that axial cross-sections could be obtained, the samples were oriented to get radial cross-sections. Samples were sectioned at 7 μm using Feather C35 microtome blades. Samples were stained with Haematoxylin & Eosin (H&E), picrosirius red (PSR) and Verhoeff’s elastin (Leica ST5010, Autostainer XL, Germany). Brightfield imaging of all stains was performed on an Aperio CS2 microscope with ImageScope software V12.3 (Leica Biosystems Imaging, Inc., Vista, California). Polarised light microscopy (PLM) was performed on the PSR-stained samples using an Olympus BX41 microscope with Ocular V2.0 software (Teledyne Photometrics, Tuscon, Arizona). Two PLM images were taken for each slice, 45° to each other, with the exposure time kept consistent across all samples.

### Registration

For the co-registration, several reference points were used, namely (i) the bifurcation, (ii) the base of the plaque in the common carotid, (iii) notable calcifications, and (iv) the 3D-printed holder. These reference points allowed the MR data to be segmented into individual volumes for each mechanically tested strip. Figure 1 shows an example of the MR data overlaid on the plaque, with the red lines denoting the imaging field of view. By knowing the width of the samples measured in ImageJ (2 mm approximately) and the slice thickness of the DTI images (0.25 mm), each strip corresponded to approximately eight MR slices.

### Data processing

#### DTI data analysis

All raw data was denoised and corrected for Gibbs ringing in MRtrix3 prior to fitting the mono-exponential tensor model in ExploreDTI (Leemans et al. 2009; Tournier 2019). From the tensor the fractional anisotropy (FA), mean diffusivity (MD), and helical angles (HA) were calculated. FA is a normalized measure of how much the eigenvalues differ and informs the degree of anisotropic diffusion within a voxel (Little and Holloway 2007). MD is the average of the eigenvalues and describes how much diffusion is occurring within a voxel. The first eigenvector (FE) was used to calculate the helical angle:

1 $$\mathrm{HA}=\mathrm{arctan}\frac{{\epsilon }_{1z}}{{\epsilon }_{1x}}$$

where ${\epsilon }_{1z}$ and ${\epsilon }_{1x}$ are the z- and x-components of the FE. The calculated HA represents the angle between the predominant direction of diffusion, the FE, and the plane normal to B_0_, the main magnetic field. Due to the presence of calcifications which are visualised as noise in DTI data and inherently bias the metrics (Farrell et al. 2010), some MR data was excluded from analysis. Specifically, regions of low signal (below the 50% percentile of the non-weighted image) and regions with high residuals from the tensor fitting (above the 99^th^ percentile) were removed. Lastly, the average MD of background PBS was used to remove the background and stray pixels were manually removed to yield the final tissue mask. After registration, as described in Sect.  2.5, 17 DTI volumes were obtained which represented the 17 mechanically tested specimens. Due to significant tissue heterogeneity, regions of the strips which were within the grips during testing were manually removed through visual inspection of images of the sample taken prior to testing and the high-resolution DIC images. Supplementary Fig. 2 shows the visual removal of MR data throughout these steps. At this point, the final tissue regions for each mechanically tested specimen were obtained and average values of FA, MD, and HA were extracted for each specimen (one mean value and standard deviation).

#### Tractography

Deterministic tractography was performed in ExploreDTI both on whole plaques as well as individual strips. For whole plaques, the following parameters were used: seed point resolution: 0.25 × 0.25 × 0.25 mm, seed FA threshold: 0.075, FA tracking threshold range: 0.075–1, MD tracking threshold range: 0-infinity, linear, planar, and spherical geometry tracking threshold range: 0–1, fibre length: 2–50 mm, angular threshold: 90°, linear interpolation, and no random perturbation of seed points. For individual specimens the same parameters were refined slightly: FA threshold (0.05), FA tracking range (0.05–1), and the fibre length (1–50 mm). This refinement allowed more fibres (at a lower FA and shorter length) to be tracked. This wasn’t necessary for the larger whole plaque but gave more detail for individual strips. Specimens were visually grouped into four groups based on tractography, as outlined in Table 1. Tracts oriented in the circumferential-radial plane, the transverse plane, represent circumferentially aligned tracts and are visualized by red-green tracts. Tracts tending towards out-of-plane alignment, or more axial alignment in the z-axis or longitudinal direction, are visualized in blue.

**Table 1:** Criteria for groupings based on morphological features visible in tractography. Circumferential alignment was determined by red-green tracts

	Criteria 1	Criteria 2	Criteria 3
Group 1	Circumferential tracts along entire gauge length on medial side		
Group 2	Circumferential tracts along entire gauge length on medial side	Circumferential alignment at one end of gauge length on luminal side	
Group 3	Circumferential tracts along entire gauge length on medial side	Thick region of plaque in middle of gauge length on luminal side	Mixed alignment covering thick region
Group 4	No clear orientation along entire gauge length, luminal or medial side		

#### Mechanical data

The cross-sectional area of each specimen was used to calculate engineering stress and the force–deformation curves after preconditioning were used to establish the stress–strain behavior of the samples. Failure was defined at the first evidence of failure (Teng et al. 2009), when the force decreased by 5%, and at this point the ultimate tensile (UT) stress and UT strain values were extracted. The final slope of the curve (collagen dominant final elastic modulus) was also calculated for each sample by taking 30 data points in length, ending before the final 20% of the total curve, and calculating the slope of the final linear region in the stress–strain curves (Whelan et al. 2019). This method allowed for the exclusion of jagged ends of the curve where sample tearing often occurred before final failure and ensured only the final slope was calculated. DIC analysis was performed on Istra4D (x64 V4.4.6.534). Evaluations were done with the following parameters: facet size: 69, 3D residuum: 10, grid spacing: 15 pixels and a low outlier tolerance. The reference frame for all DIC analysis was the frame at the end of the preconditioning cycles prior to the start of extension to failure. Engineering strain was investigated from DIC as both (1) the average strain across the gauge length on the tissue surface, called DIC strain, and (2) mean strain locally at the point of failure, called DIC local failure strain (Supplementary Fig. 3).

### Statistics

Statistical analyses were performed using GraphPad Prism (Version 8). All data was tested for normality using Shapiro–Wilk normality tests and equality of group variances using Brown-Forsythe ANOVAs. All data in this study passed normality tests. In the case of unequal group variances, Brown-Forsythe and Welch ANOVAs with Dunnett’s T3 multiple comparison tests were used, otherwise ordinary one-way ANOVAs with Tukey’s multiple comparisons were used. Pearson’s correlations were used to determine the relationship between mechanical properties and DTI metrics, with correlation coefficients (r values) < 0.3 considered weak, 0.3 < r < 0.7 considered moderate, and r > 0.7 a strong correlation. Differences between the mechanical properties of individual subjects are included in Supplementary Fig. 4. Significance was considered when p < 0.05.

## Results

**Fig. 2:**
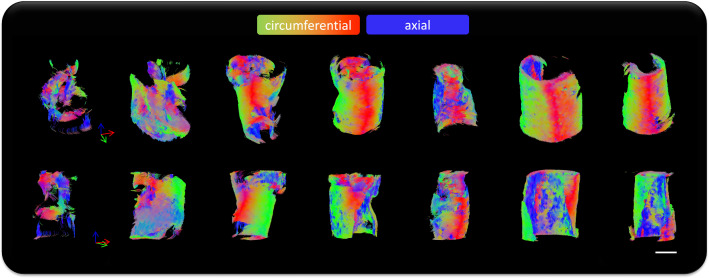
Whole plaque tractography of the seven specimens imaged and tested in this study. Red-green tracts indicate in-plane circumferential alignment whereas blue tracts represent axial out-of-plane diffusion. All specimens are scaled the same, scale bar = 5 mm.

Tractography of whole plaques can be seen in Fig. 2**.** Red-green tracts indicate in-plane circumferential alignment, while the blue tracts represent out-of-plane axial alignment. While all specimens show some degree of axial tract alignment, there are specimens which exhibit a more disorganised alignment overall, such as the first three plaques in Fig. 2. Not only is there considerable variability between different specimens, variability is also evident within individual plaques.

**Fig. 3:**
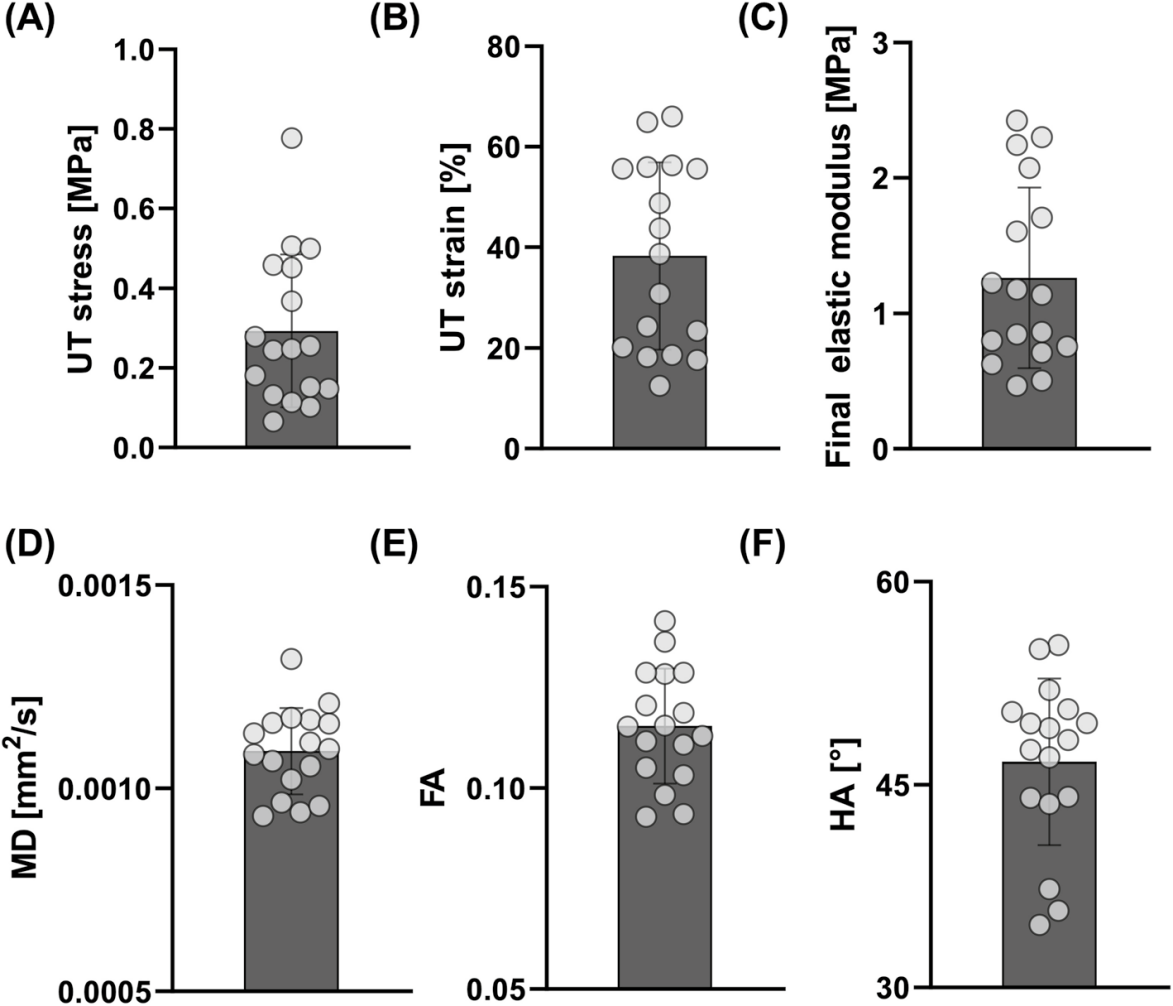
Mechanical properties and DTI-derived metrics of carotid atherosclerotic plaque strips. **A** UT stress, **B** UT strain and **C** Final elastic modulus of carotid atherosclerotic plaque strips and the corresponding DTI-derived **D** MD, **E** FA, and **F** HA

Looking at the mechanical properties and DTI-derived metrics of the 17 strips, a high degree of variability was observed, see Fig. 3. The mean UT stress and strain across the samples was 0.293 ± 0.2 MPa and 38.3 ± 19% strain, respectively. The mean final elastic modulus across all samples was 1.26 ± 0.6 MPa. The mean DTI-derived MD, FA and HA were 0.0011 ± 0.0001 mm^2^/s, 0.12 ± 0.01, and 46.7 ± 6°, respectively.

**Fig. 4:**
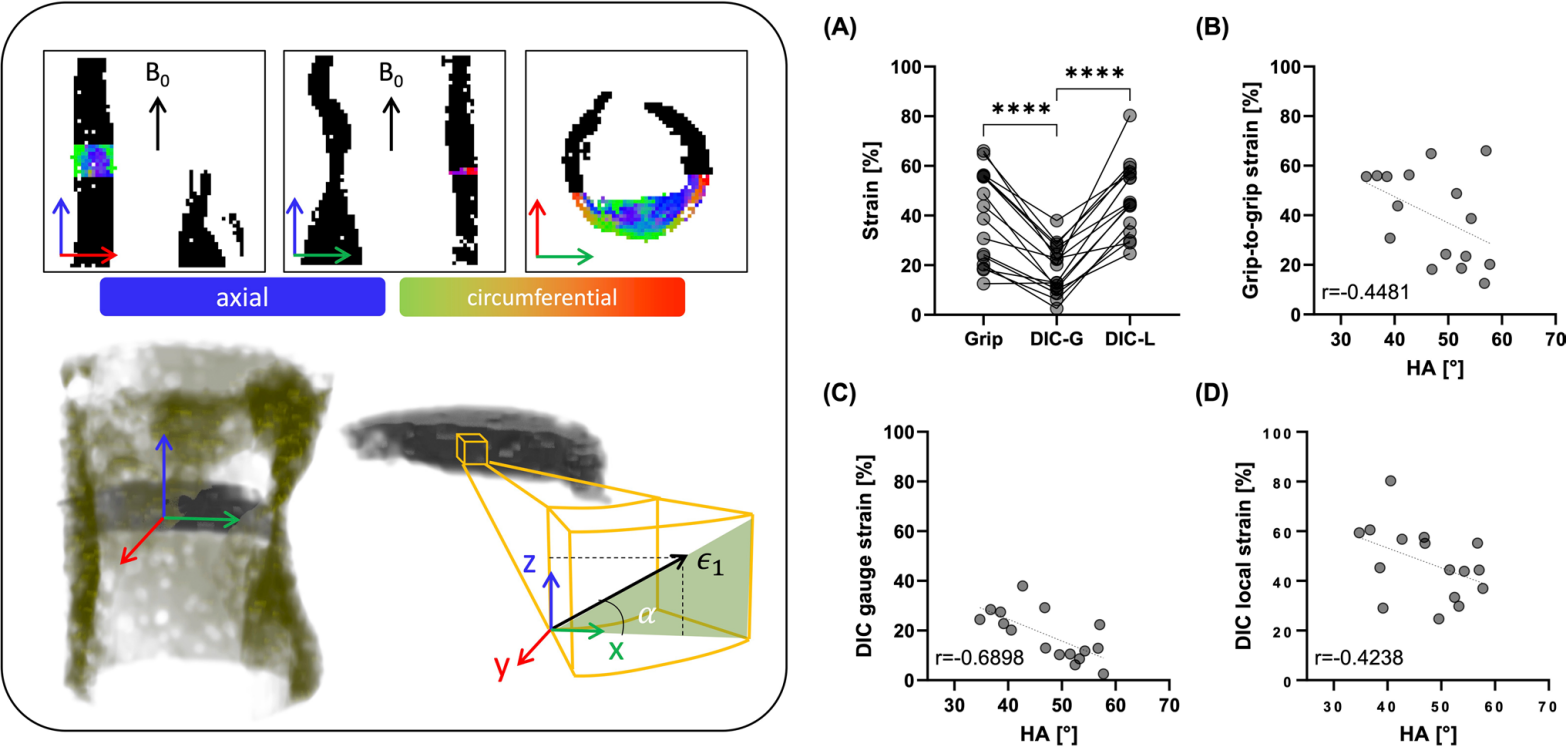
Strain measures and their correlations to DTI-derived HA. Left panel shows the three planes from MR imaging, with the whole plaque delineated in black, and the first-eigenvector map of one mechanically tested specimen overlaid. A 3D visualisation of the plaque and strip is presented below them with a schematic showing where the helical angle, alpha, is calculated by the z- and x-components of the first-eigenvector. Right panel: **A** Engineering strain from grip-to-grip (Grip), the gauge length from DIC (DIC-G), and local failure on DIC (DIC-L). Significance determined by repeated measures one-way ANOVA with Tukey’s post hoc multiple comparisons; ****p < 0.0001. Correlations between DTI-derived HA and **B** Grip-to-grip strain, **C** DIC gauge strain, and **D** DIC local strain.

The UT strain calculated from the grip-to-grip separation was significantly higher than the measured DIC strain on the tissue surface, see Fig. 4A. However, the strains seen at the local failure locations on DIC were significantly higher than the mean strain across the tissue surface. When looking at how these strains correlate to the DTI-derived HA, the DIC gauge strain demonstrated the strongest correlation, Fig. 4C, followed by grip-to-grip strain, Fig. 4B, and then the DIC local failure strain, Fig. 4D.

**Fig. 5:**
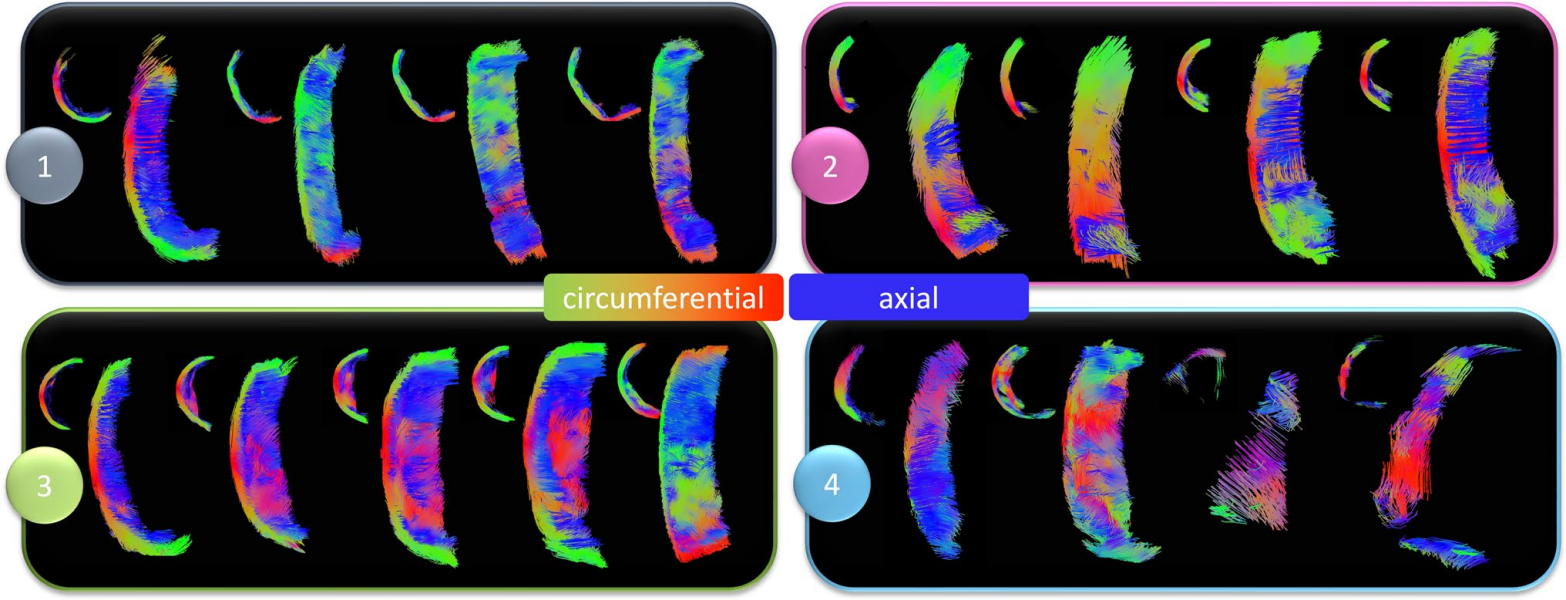
Tractography groupings of atherosclerotic plaque strips. (1) Predominantly circumferentially aligned tracts with sparce axial tracts on the luminal edge (concave side). (2) Predominantly circumferentially aligned tracts with the presence of a plaque cap shoulder – visible at the junction between fibres on the luminal side. (3) Circumferentially aligned medial tracts with large regions of mixed microstructural alignment on the luminal side and (4) overall mixed samples with no clear alignment. Red-green tracts indicate circumferential alignment, while blue is out-of-plane, axially aligned tracts.

Tractography of individual circumferentially cut strips highlighted the presence of four different microstructural alignments, see Fig. 5. While most samples showed some circumferential alignment (Fig. 5 (1–3)), n = 4 strips showed predominantly circumferential alignment with sparse axial tracts on the luminal side (Fig. 5 (1)) and n = 4 strips were circumferentially aligned with the presence of a plaque cap shoulder (Fig. 5 (2)). Figure 5 (3) shows n = 5 strips which had circumferentially aligned tracts on the more medial side and a thick mixed alignment visible on the luminal edge, and n = 4 strips with no clear alignment in Fig. 5 (4).

**Fig. 6:**
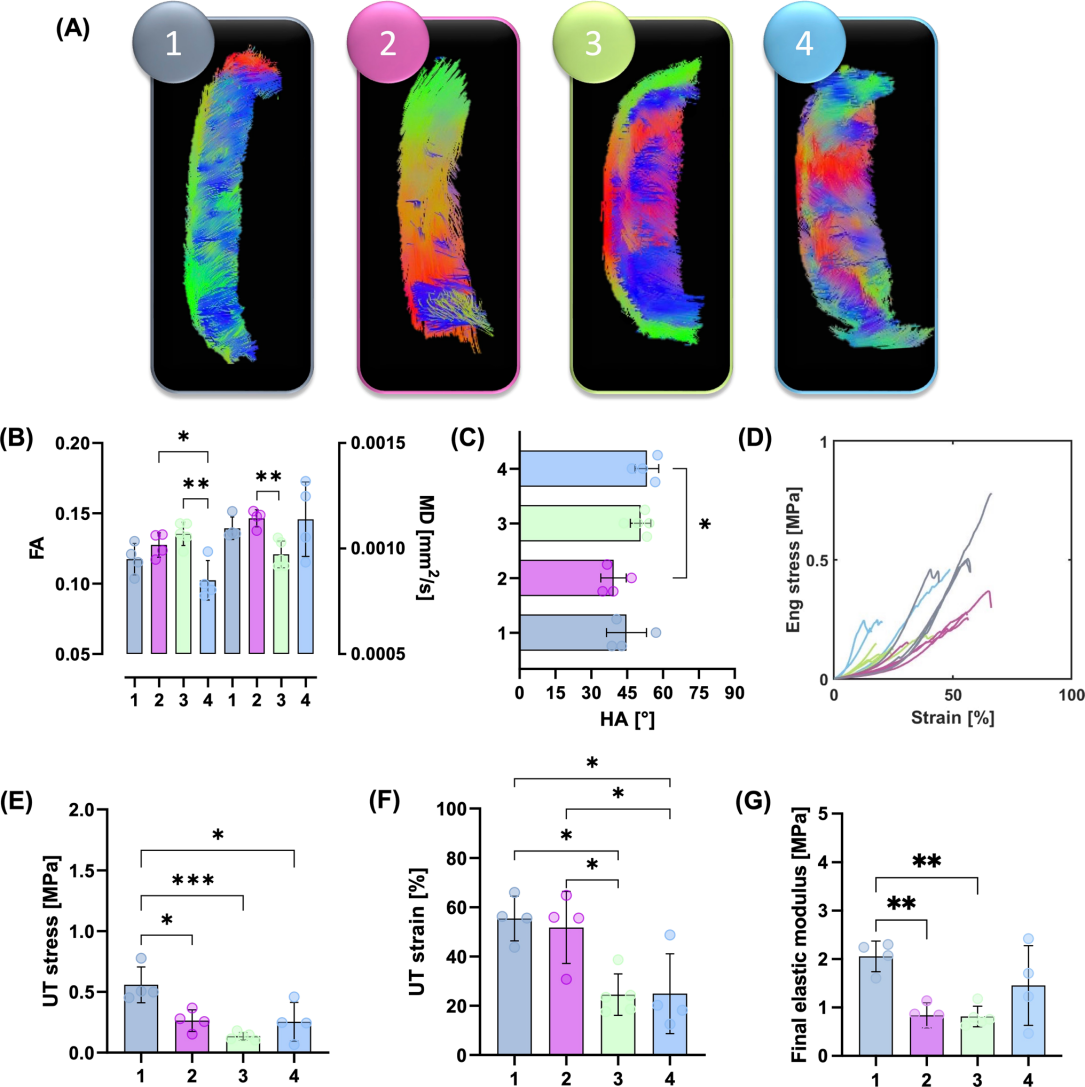
Mechanical properties of atherosclerotic plaque strips when informed by DTI-derived tractography. **A** Representative tractography of strip samples in each group.  **B** FA and MD values within these groups; FA: significance determined by an ordinary one-way ANOVA with Tukey’s post hoc multiple comparisons, **p = 0.0023, *p = 0.0237. MD: significance determined by Brown-Forsythe and Welch ANOVA with Dunnett’s T3 post hoc multiple comparisons, **p = 0.0089. **C** Mean HA for each group; significance determined by ordinary one-way ANOVA with Tukey’s post hoc multiple comparisons, *p = 0.0227. **D** Stress–strain curves for n = 17 strips, colour coded by their respective groupings. **E** UT stress; significance determined by ordinary one-way ANOVA with Tukey’s post hoc multiple comparisons, Group 1 and 2 *p = 0.0138, Group 1 and 3 ***p = 0.0005, and Group 1 and 4 *p = 0.0112. **F** UT strain; significance determined by an ordinary one-way ANOVA with Tukey’s post hoc multiple comparisons, Group 3 and 2 *p = 0.0225, Group 3 and 1 *p = 0.0145, Group 4 and 2 *p = 0.0255, and Group 4 and 1 *p = 0.0191. **G** Final elastic modulus; significance determined by Brown-Forsythe and Welch ANOVA with Dunnett’s T3 post hoc multiple comparisons, Group 1 and 2 **p = 0.0051 and Group 1 and 3 **p = 0.0053.

Using the tractography-based groupings from Table 1 and shown in Fig. 5, significant mechanical insight was uncovered, see Fig. 6. The FA in mixed Group 4 strips (0.102 ± 0.01) was significantly lower than that in Group 3 (0.135 ± 0.008) and 2 (0.128 ± 0.009) strips, see Fig. 6B. The MD in Group 3 strips (0.0009 ± 0.00006 mm^2^/s) was significantly lower than that in Group 2 strips (0.0011 ± 0.00004 mm^2^/s). The HA was only significantly different between Group 2 and 4 strips, at 39.3 ± 5.32° and 53.3 ± 4.98°, respectively (Fig. 6C).

The stress–strain curves presented in Fig. 6D illustrate the different mechanical behaviour between these strip specimen groupings. Predominantly circumferentially aligned strips in Group 1 have a significantly higher UT stress (0.559 ± 0.1 MPa), than those in Group 2 (0.264 ± 0.09 MPa), Group 3 (0.124 ± 0.02 MPa), and Group 4 (0.254 ± 0.2 MPa), see Fig. 6E. The UT strain (Fig. 6F) of Group 3 strips (24.5 ± 8.43%), was significantly lower than that of Group 2 (51.8 ± 14.6%) and Group 1 (55.5 ± 9.11%) strips. Group 4 strips also had a significantly lower UT strain (24.9 ± 16.23%) than Groups 2 and 1. Figure 6G highlights the significantly stiffer response of Group 1 strips (2.06 ± 0.3 MPa) compared to both Group 2 (0.838 ± 0.2 MPa) and Group 3 (0.815 ± 0.2 MPa).

**Fig. 7:**
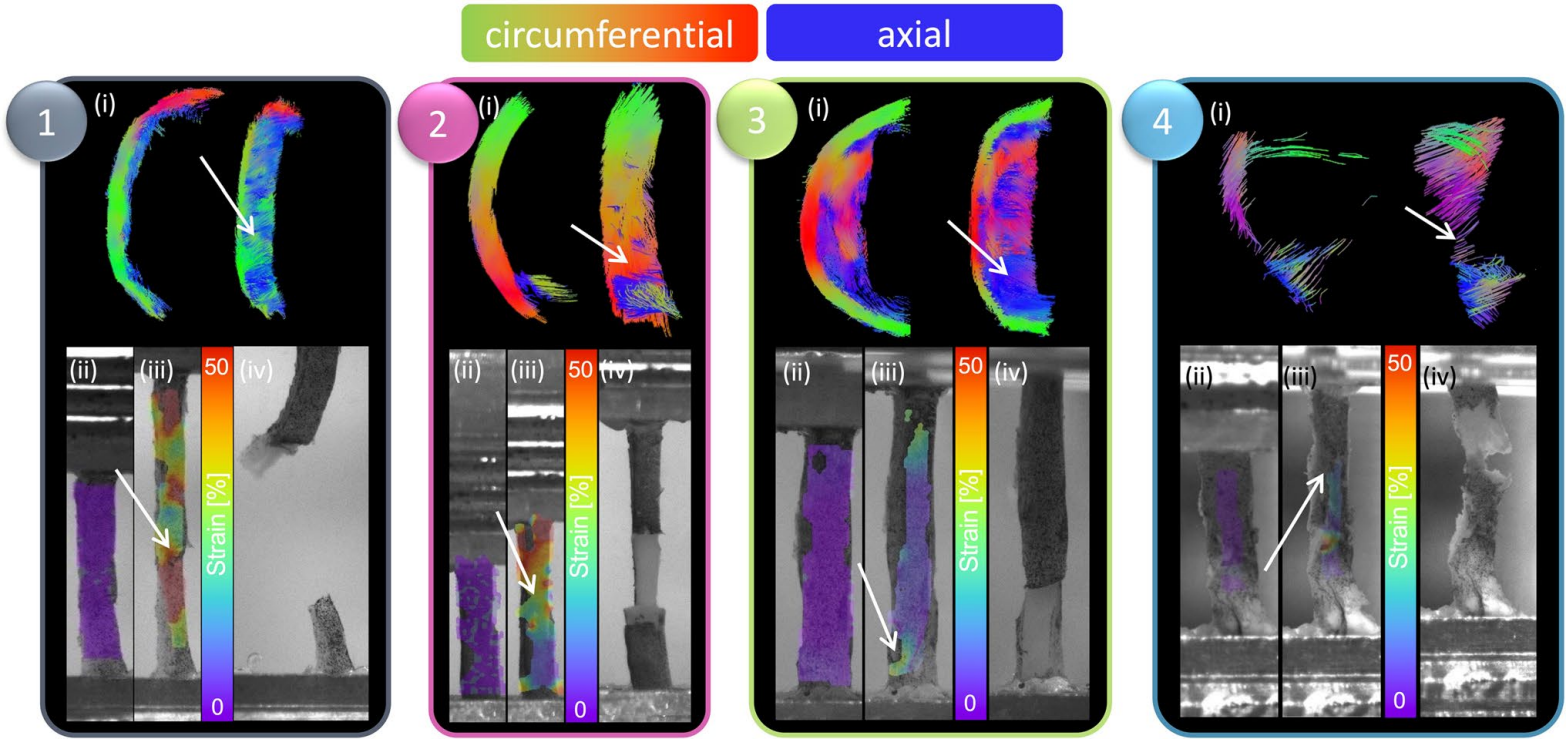
DIC strain contours and failure insights based on tractography groupings. For each grouping, (i) representative tractography is shown at the top, alongside strain contours on the DIC images at the (ii) reference frame (after preconditioning) and (iii) right before failure and a (iv) high-resolution image of the specimen right after failure. White arrows point to location of failure both on tractography and on strain maps.

DIC not only allowed for strain contours to be displayed on the tissue surface but allowed for retrospective insight into how the strips failed. Figure 7 presents strain maps on representative strips for each group. High strain (shown in red) can be seen at the point of failure in Group 1 followed by abrupt failure (Fig. 7 (1)). Specimens in Group 2 consistently failed at the junction between differing microstructures present at the plaque cap before delaminating behind the lipid core (Fig. 7 (2)). Interestingly, the specimens in Group 3 similarly showed intimal tearing, however, they delaminated through the thickness of the mixed region (Fig. 7 (3)). Specimens in Group 4 failed quite variably. In the example strip shown, the location of failure did not show the highest local strain, showing that observable higher strains are not always co-located with failure.

**Fig. 8:**
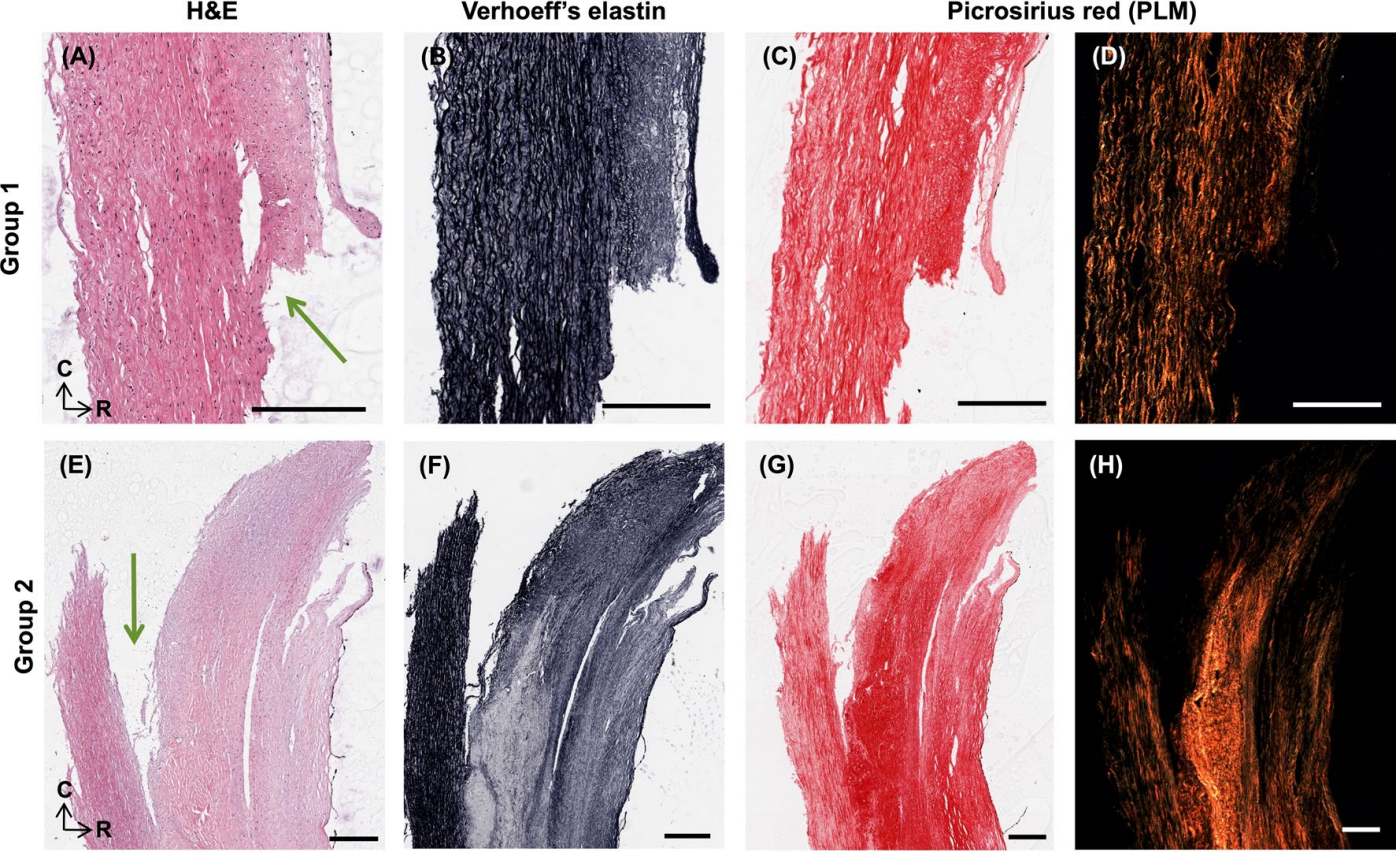
Representative histology of strips in **A**–**D** Group 1 and **E**–**H** 2. H&E, Verhoeff’s elastin, PSR and PLM are presented for each sample. Green arrows point to location of failure. Histology oriented to show axial cross-sections, moving from luminal edge towards media right to left and circumferential orientation top to bottom. All scale bars are 300 μm.

**Fig. 9:**
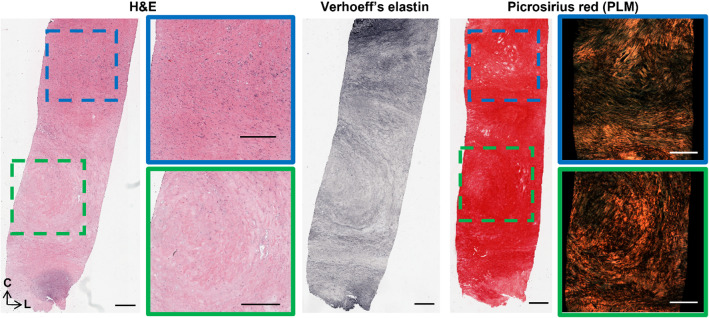
Representative histology of strips in Group 3. H&E, Verhoeff’s elastin, PSR and PLM are presented as radially sliced cross-sections: circumferential direction is top to bottom, while left to right is the longitudinal direction. Slices were taken at a depth approximately 140 μm from the luminal edge. Failure occurred at the bottom edge of the sample. All scale bars are 500 μm.

**Fig. 10:**
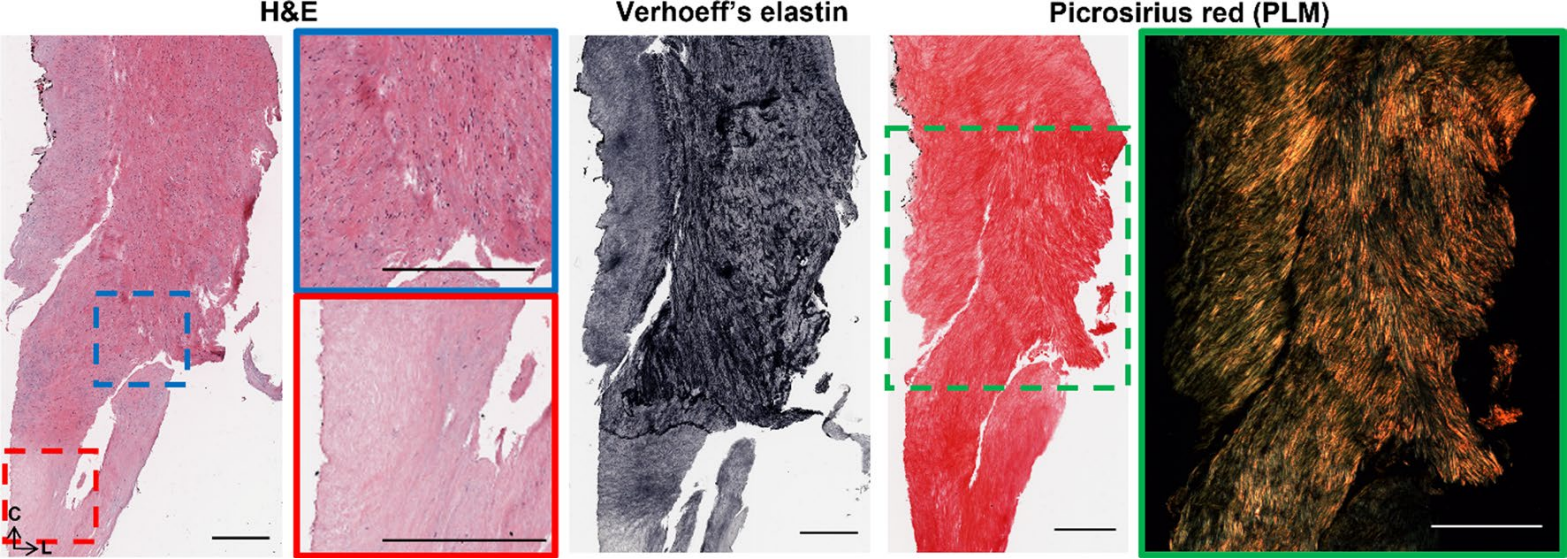
Representative histology of strips in Group 4. H&E, Verhoeff’s elastin, PSR and PLM are presented as radially sliced cross-sections: circumferential direction is top to bottom, while left to right is the longitudinal direction. Slices were taken at a depth approximately 140 μm from the luminal edge. Failure occurred from the blue box to the red box. All scale bars are 500 μm.

Representative histology of the strips shown in Fig. 7 are shown in Figs. 8, 9 and 10. Figure 8 shows axial cross-sections for both strips in both Group 1 and 2. For both groups, failure occurred at the junction between differing microstructures, as pointed out by the green arrows. In the Group 1 sample, intimal thickening can be seen in H&E and decreased elastin is also visible in the Verhoeff’s-stained cross-section. PSR and PLM highlight the circumferential arrangement of this sample, as seen in tractography. Similarly, circumferential arrangement can be seen on the more medial side (left side of image) in the Group 2 sample. However, there is a distinct delineation of differing microstructure, where failure propagated behind the lipid core.

Figure 9 similarly shows representative histology for the Group 3 specimen shown in Fig. 7. Unlike the cross-sections in Fig. 8, these are radial slices through the thickness of the plaque wall due to difficulties orienting and embedding these mechanically tested strips. This specimen failed at the bottom edge of the strip, located at the bottom of the images, at what appears to be a plaque cap shoulder. The green box points to the centre of the strip (located in the centre of the gauge length) which is acellular, low in elastin, and has disorganised collagen (seen in PLM). The blue box highlights a plaque cap shoulder which could be like the shoulder at which the sample failed. Increased cell density is seen in this region, with nuclear alignment tending to be more longitudinally oriented. Distinct longitudinally aligned collagen fibres can be seen in PLM alongside regions of disorganisation.

Lastly, Fig. 10 highlights radial cross-sections of the Group 4 specimen seen in Fig. 7. Failure delaminated down the length of the specimen, seen in Fig. 7 (4), and H&E presents the variable cell densities circumferentially. PLM shows collagen alignment tending towards circumferential but overall, quite disorganised.

## Discussion

This study used DTI-derived metrics to investigate the mechanical integrity of carotid atherosclerotic plaques. Akyildiz et al. looked at tractography-derived fibre orientation in carotid plaques and, while variable, found the predominant direction to be circumferential (Akyildiz et al. 2017). Additionally, Opriessnig et al. used tractography to visualize the circumferential alignment both of the vessel wall and the plaque cap in one cadaveric carotid artery (Opriessnig et al. 2018). When assessing the overall tractography of plaques in this study, varying degrees of alignment can be seen (Fig. 2). Variations in the microstructure can be seen both circumferentially, as well as longitudinally through the length of the plaque. These qualitative insights highlight the degree of disorganization and microstructural variation, not only between different plaques, but also within individual plaques. We have previously seen that DTI-derived metrics in arterial tissue are significantly sensitive to cell content and elastin content in similar ex vivo conditions (Tornifoglio et al. 2020; Tornifoglio et al. 2022; Shahid et al. 2017). In arterial tissue where smooth muscle cells reach lengths of 200 μm and are approximately 24% of the wall by volume and 40–60 elastic laminae, 29% by volume (O’Connell et al. 2007), maintain the highly structured framework of the tissue (Ushiki 2002), the presence or lack thereof significantly influences the diffusion within the tissue. While collagen does not directly influence the measurable anisotropic diffusion in arterial tissue, smooth muscle cells are responsible for its turnover. Therefore, as collagen and cells co-align, DTI-derived metrics are sensitive to this alignment. The tractography alignments seen in this study were investigated mechanically and histologically to see how the sensitivity of DTI to the main components of arterial tissue link to their mechanical integrity.

The well-documented variable mechanical response of carotid plaques (Maher et al. 2009; Lawlor et al. 2011; Davis et al. 2016; Mulvihill et al. 2013; Walsh et al. 2014) was also seen in this study. It is worth mentioning there is a wide range of testing parameters, such as pre-conditioning, pre-loading, and strain rates, which make comparisons challenging (Walsh et al. 2014). While testing parameters in this study follow suggested parameters (Walsh et al. 2014), the strain rate is lower than what would be experienced in vivo with instantaneous systolic pulse (Mulvihill and Walsh 2013). The UT stresses of circumferentially cut strips in this study are within the range of those determined previously (Maher et al. 2009; Lawlor et al. 2011; Mulvihill et al. 2013). More specifically, Groups 2, 3, and 4 failed at stresses lower than lightly calcified plaques (Mulvihill et al. 2013; Cunnane et al. 2015) and plaque caps with axially fibre alignment (Johnston et al. 2021). The strips in this study had no obvious evidence of calcifications and instead were fibrotic and lipid-rich, Fig. 8 (E–H) and Figs. 9–10. Group 1 strips failed at significantly higher stresses, although not quite as high as plaque caps with circumferentially aligned fibres (Johnston et al. 2021) or the isolated medial layer of carotid plaques (Teng et al. 2009). These strips were predominantly circumferentially aligned with respect to cells and collagen, although intimal thickening was evident (Fig. 8A–D). While the final elastic moduli observed in all groups in this study are higher than those reported for isolated plaque caps, they are within the ranges reported by Loree et al. of cellular atherosclerotic aortic tissue (Loree et al. 1994). As evidenced in the tractography in Fig. 5, the different groupings each demonstrated varying degrees of circumferential, axial, and mixed alignments. Interestingly, the Group 3 strips with thick mixed regions on the luminal side actually failed at lower stresses than moderately and heavily calcified plaques (Mulvihill et al. 2013; Cunnane et al. 2015). While calcifications have previously been pointed to as a vulnerable feature (Gijsen et al. 2021; Barrett et al. 2019), this finding highlights the significance of microstructural disorganization as well.

The strains for Group 1 and 2 strips are also in the range of previous studies (Mulvihill et al. 2013; Cunnane et al. 2015; Teng et al. 2009); however, Group 3 and 4 strips failed at lower strains more comparable to those of delaminated carotid plaque caps (Johnston et al. 2021). Previous studies pre-operatively used ultrasound to classify plaques as calcified, echolucent, or mixed and found no significance between groups. Only when using post-operative Fourier Transform Infrared analysis, was Mulvihill et al. able to differentiate between plaque compositions and their mechanical properties in pure shear tests (Mulvihill et al. 2013). Similarly, it was only when using tractography of individually tested atherosclerotic strips that different microstructures became apparent in this study (Fig. 5). Ultimately, these microstructures yielded significant mechanical insight into the plaque tissue. The earliest sign of progressive atherosclerosis is the thickening of the intima, classified as an American Heart Association Type III lesion (Stary et al. 1994; Sakakura et al. 2013). Group 1 strips in this study, defined as predominantly circumferential tracts with sparse axial diffusion on the luminal edge, showed signs of intimal thickening histologically (Fig. 5 (A) and Fig. 8). These samples failed at significantly higher stresses and strains than the other microstructures in this study.

Following the thickening of the intima, American Heart Association Type IV plaques or fibroatheromas can develop (Stary et al. 1995). Thin cap fibroatheromas exhibit low smooth muscle cell density in the plaque cap which overlays a lipid or necrotic core (Virmani et al. 2006); however, these atheromas often fail to narrow the vascular lumen despite thickening the arterial wall (Glagov et al. 1971). Both Groups 2 and 3 in this study exhibited the circumferential alignment known to be present in the medial layers of healthy arterial walls (Fig. 5B and C), but also showed signs of more advanced plaque development. Group 2 strips were similar in microstructure to Group 1 but had the distinct presence of a plaque cap shoulder. Tractography was capable of visualising the plaque cap and also showed a circumferential alignment of the cap, see arrow in Fig. 7 (2), which was corroborated by the PLM histological images seen in Fig. 8. Circumferential alignment is clear on both the luminal and medial edges, and surrounds an elastin-poor, disorganised (with respect to cell and collagen content) region. There also appears to be evidence of cholesterol crystals, which are believed to arise from cellular apoptosis (Sakakura et al. 2013). Mechanically, these strips appeared to initially strain similar to Group 1 strips. When looking to the DIC images, the circumferentially aligned regions are bearing the load, until ultimately failure occurs at a significantly lower stress at the junction between the circumferential medial regions and the plaque cap shoulder (Fig. 7 (2)). Group 3 strips showed varying degrees of alignment in the plaque cap, but all show a thick, mixed region between the cap and the medial layers of the plaque. While both Groups 2 and 3 failed on the luminal edge of the plaque, Group 3 failed through the mixed region whereas Group 2 strips delaminated behind the lipid core – seen both in the DIC images and histologically (Fig. 7 (2 and 3) and Fig. 8). Mechanically, Group 3 strips failed at significantly lower stresses and strains than both Groups 1 and 2 – identifying them as the most vulnerable microstructure of those seen in this study (Fig. 6E and F). When looking histologically, it becomes clear that Group 3 strips also failed at the plaque cap shoulder – where there is a distinct difference in cell density and collagen orientation (Fig. 9). The mixed microstructures in Group 4, unsurprisingly, show highly variable mechanical properties and a highly disordered microstructure via tractography (Fig. 5 (4)) and histology (Fig. 10). Together, these results suggest that under the same physiological conditions, plaque tissue with a microstructural resembling that of Group 3 strips would be the most vulnerable to rupture. Conversely, a microstructural alignment mimicking that in Group 1 would be the least vulnerable and more stable – suggesting intervention may not be needed.

Another interesting finding from this work highlighted differences in the strain across the tissue. The strain calculated from the grip-to-grip separation was considerably higher than the strain across the tissue surface measured using DIC. However, the local failure strains on the tissue surface were significantly higher than these mean strains on the tissue surface. Previous work has shown that strain, rather than stress, might be a better indicator for plaque cap vulnerability (Davis et al. 2016; Johnston et al. 2021). The work presented here shows that the strain at local regions of plaque rupture might be significantly higher than the overall strain of the tissue.

While this study provides novel insights between a clinically relevant imaging technique and mechanical characteristics of human plaques, there are some limitations to the study. Firstly, both biological sample and tested strip numbers are low. The imaging field of view confined usable strip specimens to a localised region and samples which could not be registered back to this field of view were excluded, as well as samples which failed near the grips. Despite these challenges, the limited sample numbers show promise given their significant differences. With increased plaque samples and individual strips, the groupings used in this study could be applied across entire plaques to determine the plaque’s vulnerability, rather than strips. DIC strain measures were not used after initial intimal tearing as the tissue surface with the speckle pattern was disconnected from the more medial layer which continued to bear load. Additionally, while the lengthy scan time allowed for in depth non-invasive characterisation, it will also lead to some tissue degradation. Despite this, all samples were subjected to the same scan times and their relative differences can be compared. Future use of echo planar imaging would speed up acquisition significantly and limit potential degradation. In the future it would be advantageous to utilise bi-axial testing or inflation testing of plaque tissue to gain more physiological insight into the mechanical response of the tissue. The incorporation of an MRI compatible bioreactor which allows for imaging before and after testing would yield novel insights into how the underlying microstructure is changing under loading and ultimately DTI-derived metrics, such as the helical angle, could be incorporated into finite element models (Pierce et al. 2013; Stadelmann et al. 2018).

The groupings in this study were visually determined based on local microstructural features visible through tractography. Although no singular quantitative DTI-derived metric was feasible to group individual plaque strips, visual inspection of DTI-derived tractography still has its benefits. By increasing sample numbers in future work, machine learning could be implemented to identify key features, such as the plaque cap, and their respective alignment (or lack thereof). Additionally, expanding this to whole plaques, rather than strips, will undoubtably increase intrasample variability – making a singular quantitative metric elusive, whereas local visualisation of the microstructure and key morphologies is more beneficial. 

It would be naïve to not address the hurdle of clinical translation for a DTI sequence at the carotid bifurcation. Acquisition challenges stemming from cardiac and respiratory motion and scan duration mean translation is not a trivial task. The idealized ex vivo set-up in this study allowed for a high-resolution, 0.250 × 0.250 × 0.250 mm, look at arterial microstructure. The only in vivo arterial DTI study conducted to date achieved an in-plane resolution of 0.55 × 0.55 mm, interpolated further to 0.2 × 0.2 mm (Opriessnig et al. 2016). To truly gauge the usefulness of this technique in the clinic, future studies should investigate the attainable in vivo resolutions and if the signal-to-noise ratio achieved is adequate. However, this work provides the fundamental insights into DTI-derived metrics and the mechanical insight they can yield for future translational studies. Additionally, a number of in vivo studies have used diffusion weighted imaging to investigate plaque components (Alex et al. 2022; Kim et al. 2011; Kim et al. 2021; Millon et al. 2013; Xie et al. 2014; Lopez Gonzalez, et al. 2016; Young et al. 2010; Zhang et al. 2017), already establishing the usefulness of measuring water diffusion within these tissues. Extending these acquisitions to incorporate directionality, while not trivial, has the potential to better ascertain plaque vulnerability due to the direct link to mechanical integrity. While achieving a singular quantitative metric which ultimately dictates a plaque’s vulnerability is far-fetched, it is possible that the microstructural insight observed via DTI-tractography could aid in clinical decision making for lower grade stenosed or asymptomatic patients. More so, while longitudinal imaging is not currently in place for atherosclerosis patients, scenarios where patients are monitored long term – such as aneurysms – could benefit from non-invasive, mechanically sensitive insight.

## Conclusion

In this study, fresh human atherosclerotic plaques from endarterectomy surgeries were imaged ex vivo with a DTI sequence, mechanically tested, and investigated histologically. For the first time, this work identified a non-invasive MR imaging technique which could yield microstructural insight into atherosclerotic plaques and could ultimately be used to identify microstructures more at risk of rupture. These novel findings have the potential to drive continued research in non-invasive imaging techniques linked with mechanical characterisation to better identify plaque vulnerability.

### Supplementary Information

Below is the link to the electronic supplementary material.

Supplementary file1 (DOCX 4020 kb)

## References

[CR1] Akyildiz AC (2017). 3D fiber orientation in atherosclerotic carotid plaques. J Struct Biol.

[CR2] Alex A (2022). Role of diffusion - weighted imaging in carotid plaque vulnerability assessment. Egypt J Radiol Nucl Med.

[CR3] Azuma M (2020). Characterization of carotid plaque components by quantitative susceptibility mapping. Am J Neuroradiol.

[CR4] Barrett HE, Van der Heiden K, Farrell E, Gijsen FJH, Akyildiz AC (2019). Calcifications in atherosclerotic plaques and impact on plaque biomechanics. J Biomech.

[CR5] Born GVR, Richardson P (1990). Mechanical properties of human atherosclerosis. Pathobiol Human Atheroscler Plaques.

[CR6] Cahalane RM (2018). Relating the mechanical properties of atherosclerotic calcification to radiographic density: A nanoindentation approach. Acta Biomater.

[CR7] Cahalane RM, Walsh MT (2021). Nanoindentation of calcified and non-calcified components of atherosclerotic tissues. Exp Mech.

[CR8] Clarke SE, Hammond RR, Mitchell JR, Rutt BK (2003). Quantitative assessment of carotid plaque composition using multicontrast MRI and registered histology. Magn Reson Med.

[CR9] Creane A (2012). A remodelling metric for angular fibre distributions and its application to diseased carotid bifurcations. Biomech Model Mechanobiol.

[CR10] Cunnane EM (2015). Mechanical, biological and structural characterization of human atherosclerotic femoral plaque tissue. Acta Biomater.

[CR11] Cunnane EM (2016). Mechanical properties and composition of carotid and femoral atherosclerotic plaques: A comparative study. J Biomech.

[CR12] Davis LA (2016). Characterization of fracture behavior of human atherosclerotic fibrous caps using a miniature single edge notched tensile test. Acta Biomater.

[CR13] Ebenstein DM, Coughlin D, Chapman J, Li C, Pruitt LA (2009). Nanomechanical properties of calcification, fibrous tissue, and hematoma from atherosclerotic plaques. J Biomed Mater Res - Part A.

[CR14] Elhfnawy AM, Volkmann J, Schliesser M, Fluri F (2019). Symptomatic vs asymptomatic 20–40% internal carotid artery stenosis: does the plaque size matter?. Front Neurol.

[CR15] Farrell JAD (2010). Effects of SNR on the accuracy and reproducibility of DTI-derived fractional anisotropy, mean diffusivity, and principal eigenvector measurements at 1.5T. J Magn Reson.

[CR16] Ghasemi M, Nolan DR, Lally C (2020). Assessment of mechanical indicators of carotid plaque vulnerability: Geometrical curvature metric, plaque stresses and damage in tissue fibres. J Mech Behav Biomed Mater.

[CR17] Gijsen FJH (2021). Morphometric and mechanical analyses of calcifications and fibrous plaque tissue in carotid arteries for plaque rupture risk assessment. IEEE Trans Biomed Eng.

[CR18] Glagov S, Weisenberg E, Zarins CK, Stankunavicius R, Kolettis G (1971). Compensatory enlargement of human atherosclerotic coronary arteries. N Engl J Med.

[CR19] Haque MN, Azam MS, Sarwar MG (2022). Status of carotid artery atherosclerosis among the ischemic stroke patients. Med Res Chronicals.

[CR20] Harteveld A (2016). Quantitative intracranial atherosclerotic plaque characterization at 7T MRI: an ex vivo study with histologic validation. Am J Neuroradiol.

[CR21] Holm Nielsen S (2020). Exploring the role of extracellular matrix proteins to develop biomarkers of plaque vulnerability and outcome. J Intern Med.

[CR22] Holzapfel GA, Sommer G, Regitnig P (2004). Anisotropic mechanical properties of tissue components in human atherosclerotic plaques. J Biomech Eng.

[CR23] Huang C (2016). Ultrasound-based carotid elastography for detection of vulnerable atherosclerotic plaques validated by magnetic resonance imaging. Ultrasound Med Biol.

[CR24] Jiang P (2020). Association between carotid bifurcation geometry and atherosclerotic plaque vulnerability: a Chinese atherosclerosis risk evaluation study. Arterioscler Thromb Vasc Biol.

[CR25] Johnston RD, Gaul RT, Lally C (2021). An investigation into the critical role of fibre orientation in the ultimate tensile strength and stiffness of human carotid plaque caps. Acta Biomater.

[CR26] Karmonik C, Basto P, Morrisett JD (2006). Quantification of carotid atherosclerotic plaque components using feature space analysis and magnetic resonance imaging. Annu. Int Conf IEEE Eng Med Biol - Proc.

[CR27] Kim S-E (2011). In vivo and ex vivo measurements of the mean ADC values of lipid necrotic core and hemorrhage obtained from diffusion weighted imaging in human atherosclerotic plaques. J Magn Reson Imaging.

[CR28] Kim S-E (2021). Differentiation of symptomatic and asymptomatic carotid intraplaque hemorrhage using 3D high-resolution diffusion-weighted stack of stars imaging. NMR Biomed.

[CR29] Kolodgie FD (2017). High-risk carotid plaque: lessons learned from histopathology. Semin Vasc Surg.

[CR30] Lawlor MG, O’Donnell MR, O’Connell BM, Walsh MT (2011). Experimental determination of circumferential properties of fresh carotid artery plaques. J Biomech.

[CR31] Leemans A, Jeurissen B, Sijbers J, Jones DK (2009). ExploreDTI: a graphical toolbox for processing, analyzing, and visualizing diffusion MR data. Proc Int Soc Magnetic Resonance Med.

[CR32] Lendon CL, Davies MJ, Richardson PD, Born GVR (1993). Testing of small connective tissue specimens for the determination of the mechanical behaviour of atherosclerotic plaques. J Biomed Eng.

[CR33] Levene CI, Poole JC (1962). The collagen content of the normal and atherosclerotic human aortic intima. Br J Exp Pathol.

[CR34] Li Z (2017). Intravascular ultrasound elastography analysis of the elastic mechanical properties of atherosclerotic plaque. Int J Cardiovasc Imaging.

[CR35] Little DM, Holloway RG (2007). Diffusion tensor imaging. Neurology.

[CR36] Lopez Gonzalez MRR (2016). Atherosclerotic carotid plaque composition: a 3T and 7T MRI-histology correlation study. J Neuroimag.

[CR37] Loree HM, Grodzinsky AJ, Park SY, Gibson LJ, Lee RT (1994). Static circumferential tangential modulus of human atherosclerotic tissue. J Biomech.

[CR38] Maher E (2009). Tensile and compressive properties of fresh human carotid atherosclerotic plaques. J Biomech.

[CR39] Mekkaoui C (2018). Myocardial scar delineation using diffusion tensor magnetic resonance tractography. J Am Heart Assoc.

[CR40] Meletta R (2015). Ex vivo differential phase contrast and magnetic resonance imaging for characterization of human carotid atherosclerotic plaques. Int J Cardiovasc Imaging.

[CR41] Millon A (2013). High-resolution magnetic resonance imaging of carotid atherosclerosis identifies vulnerable carotid plaques. J Vasc Surg.

[CR42] Morrisett J (2003). Discrimination of components in atherosclerotic plaques from human carotid endarterectomy specimens by magnetic resonance imaging ex vivo. Magn Reson Imag.

[CR43] Müller-Schweinitzer E (2009). Cryopreservation of vascular tissues. Organogenesis.

[CR44] Mulvihill JJ (2013). Mechanical, biological and structural characterization of in vitro ruptured human carotid plaque tissue. Acta Biomater.

[CR45] Mulvihill JJ, Walsh MT (2013). On the mechanical behaviour of carotid artery plaques: the influence of curve-fitting experimental data on numerical model results. Biomech Model Mechanobiol.

[CR46] Nakao Y (2021). Plaque characterization with computed tomography angiography based on a diluted-contrast injection protocol. Intern Med.

[CR47] Naylor AR (2018). Editor’s choice – management of atherosclerotic carotid and vertebral artery disease: 2017 Clinical practice guidelines of the European Society for Vascular Surgery (ESVS). Eur J Vasc Endovasc Surg.

[CR48] O’Connell MK (2007). The three-dimensional micro- and nanostructure of the aortic medial lamellar unit measured using 3D confocal & electron microscopy imaging. Matrix Biol.

[CR49] Opriessnig P, Silbernagel G, Krassnig S, Reishofer G (2018). Magnetic resonance microscopy diffusion tensor imaging of collagen fibre bundles stabilizing an atherosclerotic plaque of the common carotid artery. Eur Heart J.

[CR50] Opriessnig P, Mangge H, Stollberger R, Deutschmann H, Reishofer G (2016). In vivo cardiovascular magnetic resonance of 2D vessel wall diffusion anisotropy in carotid arteries. J Cardiovasc Magn Reson.

[CR51] Pierce DMDM, Ricken T, Holzapfel GAG (2013). Modeling sample/patient-specific structural and diffusional responses of cartilage using DT-MRI. Int J Numer Method Biomed Eng.

[CR52] Rueden CT (2017). Image J2: ImageJ for the next generation of scientific image data. BMC Bioinformatics.

[CR53] Saba L (2019). Imaging biomarkers of vulnerable carotid plaques for stroke risk prediction and their potential clinical implications. Lancet Neurol.

[CR54] Sakakura K (2013). Pathophysiology of atherosclerosis plaque progression. Hear Lung Circ.

[CR55] Shahid SS, Gaul RT, Kerskens C, Flamini V, Lally C (2017). Quantifying the ultrastructure of carotid arteries using high-resolution micro-diffusion tensor imaging - comparison of intact versus open cut tissue. Phys Med Biol.

[CR56] Sherebrin MH, Bernans HA, Roach MR (1987). Extensibility changes of calcified soft tissue strips from human aorta. Can J Physiol Pharmacol.

[CR57] Shinnar M (1999). The diagnostic accuracy of ex vivo mri for human atherosclerotic plaque characterization. Arterioscler Thromb Vasc Biol.

[CR58] Stadelmann MA (2018). Integrating MRI-based geometry, composition and fiber architecture in a finite element model of the human intervertebral disc. J Mech Behav Biomed Mater.

[CR59] Stary HC (1994). A definition of initial, fatty streak, and intermediate lesions of atherosclerosis. Circulation.

[CR60] Stary HC (1995). A definition of advanced types of atherosclerotic lesions and a histological classification of atherosclerosis. Circulation.

[CR61] Stoeck CT (2021). Cardiovascular magnetic resonance imaging of functional and microstructural changes of the heart in a longitudinal pig model of acute to chronic myocardial infarction. J Cardiovasc Magn Reson.

[CR62] Subban V, Raffel OC (2020). Optical coherence tomography: fundamentals and clinical utility. Cardiovasc Diagnosis Therapy.

[CR63] Teng Z, Tang D, Zheng J, Woodard PK, Hoffman AH (2009). An experimental study on the ultimate strength of the adventitia and media of human atherosclerotic carotid arteries in circumferential and axial directions. J Biomech.

[CR64] Tornifoglio B (2020). Diffusion tensor imaging and arterial tissue: establishing the influence of arterial tissue microstructure on fractional anisotropy, mean diffusivity and tractography. Sci Rep.

[CR65] Tornifoglio B, Stone AJ, Kerskens C, Lally C (2022). Ex vivo study using diffusion tensor imaging to identify biomarkers of atherosclerotic disease in human cadaveric carotid arteries. Arterioscler Thromb Vasc Biol.

[CR66] Tournier JD (2019). MRtrix3: A fast, flexible and open software framework for medical image processing and visualisation. Neuroimage.

[CR67] Toussaint JF, Southern JF, Fuster V, Kantor HL (1997). Water diffusion properties of human atherosclerosis and thrombosis measured by pulse field gradient nuclear magnetic resonance. Arterioscler Thromb Vasc Biol.

[CR68] Truong M (2021). Classifications of atherosclerotic plaque components with T1 and T2* mapping in 11.7 T MRI. Eur J Radiol Open.

[CR69] Ushiki T (2002). Collagen fibers, reticular fibers and elastic fibers A comprehensive understanding from a mophological viewpoint. Arch Histol Cytol.

[CR70] van Soest G, Marcu L, Bouma BE, Regar E (2017). Intravascular imaging for characterization of coronary atherosclerosis. Curr Opin Biomed Eng.

[CR71] Virmani R, Burke AP, Farb A, Kolodgie FD (2006). Pathology of the vulnerable plaque. J Am Coll Cardiol.

[CR72] Walsh MT (2014). Uniaxial tensile testing approaches for characterisation of atherosclerotic plaques. J Biomech.

[CR73] Wang J, Wang L, Shen Y, Gong X, Ju Y (2022). Relationship between carotid artery angle and plaque morphology in acute cerebral infarction patients. Neurologist.

[CR74] Warlow C (1991). MRC european carotid surgery trial: interim results for symptomatic patients with severe (70–99%) or with mild (0–29%) carotid stenosis. Lancet.

[CR75] Warlow C, Farrell B, Fraser A, Sandercock P, Slattery J (1998). Randomised trial of endarterectomy for recently symptomatic carotid stenosis: final results of the MRC European Carotid Surgery Trial (ECST). Lancet.

[CR76] Wasserman BA, Wityk RJ, Trout HH, Virmani R (2005). Low-grade carotid stenosis: Looking beyond the lumen with MRI. Stroke.

[CR77] Whelan A (2019). Collagen fibre orientation and dispersion govern ultimate tensile strength, stiffness and the fatigue performance of bovine pericardium. J Mech Behav Biomed Mater.

[CR78] Xie Y (2014). High resolution 3D diffusion cardiovascular magnetic resonance of carotid vessel wall to detect lipid core without contrast media. J Cardiovasc Magn Reson.

[CR79] Yabushita H (2002). Characterization of human atherosclerosis by optical coherence tomography. Circulation.

[CR80] Young VE (2010). Diffusion-weighted magnetic resonance imaging for the detection of lipid-rich necrotic core in carotid atheroma in vivo. Neuroradiology.

[CR81] Zhang Q, Coolen BF, Versluis MJ, Strijkers GJ, Nederveen AJ (2017). Diffusion-prepared stimulated-echo turbo spin echo (DPsti-TSE): An eddy current-insensitive sequence for three-dimensional high-resolution and undistorted diffusion-weighted imaging. NMR Biomed.

